# A searchable personal health records framework with fine-grained access control in cloud-fog computing

**DOI:** 10.1371/journal.pone.0207543

**Published:** 2018-11-29

**Authors:** Jin Sun, Xiaojing Wang, Shangping Wang, Lili Ren

**Affiliations:** School of Science, Xi’an University of Technology, Xi’an, Shaanxi, China; King Abdulaziz University, SAUDI ARABIA

## Abstract

Fog computing can extend cloud computing to the edge of the network so as to reduce latency and network congestion. However, existing encryption schemes were rarely used in fog environment, resulting in high computational and storage overhead. Aiming at the demands of local information for terminal device and the shortcomings of cloud computing framework in supporting mobile applications, by taking the hospital scene as an example, a searchable personal health records framework with fine-grained access control in cloud-fog computing is proposed. The proposed framework combines the attribute-based encryption (ABE) technology and search encryption (SE) technology to implement keyword search function and fine-grained access control ability. When keyword index and trapdoor match are successful, the cloud server provider only returns relevant search results to the user, thus achieving a more accurate search. At the same time, the scheme is multi-authority, and the key leakage problem is solved by dividing the user secret key distribution task. Moreover, in the proposed scheme, we securely outsource part of the encryption and decryption operations to the fog node. It is effective both in local resources and in resource-constrained mobile devices. Based on the decisional *q*-parallel bilinear Diffie-Hellman exponent (*q*-DBDHE) assumption and decisional bilinear Diffie-Hellman (DBDH) assumption, our scheme is proven to be secure. Simulation experiments show that our scheme is efficient in the cloud-fog environment.

## 1 Introduction

With the promotion of new medical reform policies and the rapid development of medical information, Electronic Medical Record (EMR) [1] has become an inevitable outcome of network information technology in the medical field. An EMR is an electronic patient record of a specific system that is created, stored and used electronically. The results of the patient’s diagnosis and treatment results can be transmitted through the hospital’s computer network or the health card (optical card and IC card). The sharing of information resources brings great convenience to medical care. Unlike EMR, Personal Health Records (PHR) [2] are health information created and managed by patients themselves through the Internet. With the development of cloud computing, patients upload PHR files to cloud servers through mobile devices, which saves local storage space and expands information sharing.

**Fig 1:**
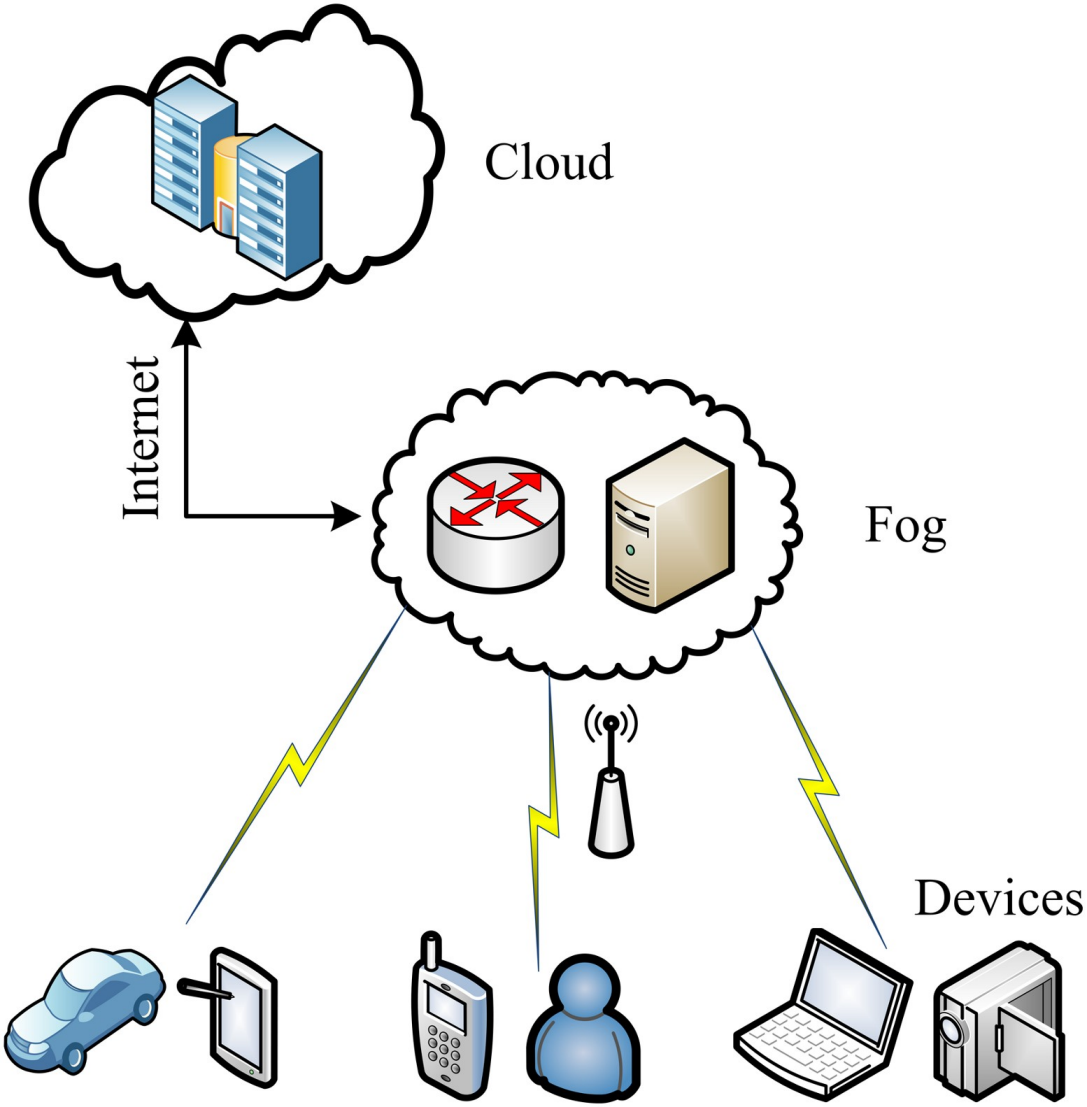
Fog computing service structure.

A large amount of data is stored in the cloud, making traditional clouds difficult to meet the current needs. The massive increase in stored data would not only cause great pressure on the cloud, but also lead to network congestion and transmission delays. For example, large enterprises need to pay for expensive bandwidth if they completely relies on the cloud for complex data processing; some requirements require timely response, such as payment links, car avoidance technologies involved in autonomous driving, and even when providing emergency medical services, data delays or cloud network failures can have serious consequences that cannot be measured. In order to solve the above problems, Bonomi F et al. of the US company Cisco first proposed the concept of fog computing in 2012 [3]. Fog computing is an extension of cloud computing and is a service computing paradigm for paravirtualized frameworks [4,5]. The fog server is set between the cloud server and the IoT devices, so as to the storage and calculation of data are transferred as much as possible to the fog servers. So, the fog computing helps to reduce the workload of the cloud server and improve the efficiency of the entire system. The fog computing service framework is shown in Fig 1.

When sensitive data are outsourced to fog nodes which are similar to cloud platform, the data security and privacy concerns still impede the adoption of fog computing as data owners lose the physical control over their data in fog nodes or cloud. Semi-trusted cloud servers may leak and tamper with PHR information or non-authorized users steal sensitive patient information for commercial benefit. If sensitive information is used by unauthorized users or third parties, the doctor may obtain a wrong medical record, resulting in misdiagnosis. How to solve the security and privacy issues in the PHR system has become one of the most important challenge.

Encryption technology is the most critical technology to ensure information security. Goyal et al. [6] formulated the ABE into two types: the ciphertext-policy attribute-based encryption (CP-ABE) and the key-policy attribute-based encryption (KP-ABE). CP-ABE is considered one of the most appropriate encryption methods to achieve fine-grained access control. This approach allows the data owner to perform access control by setting the access structure. Due to the one-to-many communication characteristics of the ABE system, flexible access control encryption schemes are being proposed. In 2010, Ibraimi et al. [7] applied ABE to PHR security management to achieve flexible access control. However, this scheme did not give concrete proof of security. In 2013, Li et al. [8] used attribute-based encryption technology to encrypt PHR files of patients, achieving scalability and fine-grained access control for PHR. Unlike other solutions, this scheme supports multiple data owner application scenarios. Compared to the traditional single-authority CP-ABE schemes, the attributes come from different attribute authorities in the multi-authority CP-ABE schemes. In addition, it does not cause single point of failure and key leakage, which makes the multi-authority CP-ABE schemes more practical in cloud-fog computing.

In addition to data security issues, supporting outsourced partial computing operations and efficient searching of encrypted data are also an important feature in practical applications.

### 1.1 Related work

In this section, we discuss the related work of this article.

#### 1.1.1 Multi-authority ABE

A single authority attribute encryption scheme manages a large number of user attribute sets by only one attribute authority, which easily causes network congestion and reduces system efficiency. This does not meet the actual work needs. In order to improve the efficiency of the single attribute authority, multiple authority ABE schemes have been proposed. In 2007, Chase et al. [9] introduced the first multi-authority ABE scheme. Independent authority supervise attributes and distribute keys to improve the security of the key. However, the encryption algorithm of the scheme is not flexible enough. In 2013, Yang et al. [10] proposed a multi-authority cloud storage data access control (DAC-MACS) scheme. The partial decryption calculation is outsourced to the server by a token-based decryption method, and the scheme also supports instant attribute revocation. However, the global certification authority has a huge amount of bilinear calculations. Subsequently, some multi-authority large-universe ABE schemes [11,12] have been proposed. In 2018, Zhang et al. [13] proposed a multi-authority CP-ABE scheme with white box tracking. The access policy can be expressed as any monotonous access structure, and the ciphertext size grows linearly with the rows of the access matrix.

#### 1.1.2 Keyword search over encrypted data

Searchable encryption (SE) can be classified into two types: symmetric encryption with keyword search (SEKS) and public key encryption with keyword search (PEKS). There are some attempts to combine data encryption and searchable encryption to ensure the security of uploading to the cloud. In 2004, Boneh et al. [14] proposed the first concept of PEKS. However, the server has a large computational overhead during the matching process between the trapdoor and the index. Then, search schemes [15–19] with different characteristics have been proposed. In 2017, Cui et al. [20] proposed a keyword search encryption scheme that supports effective revocation in cloud computing. At the same time, it supports certifiable keyword search and effective user revocation to meet the application scenarios of multiple data owners and data users. To the best of our knowledge, there is no searchable encryption scheme designed for cloud-fog environments currently.

#### 1.1.3 Outsourced ABE

In ABE, aim to reduce the local computation cost, outsourcing complex operations to a cloud server becomes an important and popular problem. In 2011, Green et al. [21] constructed the outsourced decryption ABE scheme in order to save the local computing time. The user did not require bilinear pairing operations during the decryption phase. But this scheme can not guarantee the correctness of the transformed key. In 2016, Wang et al. [22] proposed a verifiable outsourced attribute encryption scheme based on dual-system encryption technology and composite order bilinear group, it is less efficient. Recently, In 2018, Jiang et al. [23] proposed a revocable outsourcing attribute based encryption scheme. The storage service manager distributes the attribute key for the user through the binary status tree, thereby implementing user revocation and attribute revocation. There are several applications of outsourced ABE in [24–26].

### 1.2 Our contribution

In this article, we propose a searchable personal health records framework with fine-grained access control in cloud-fog computing. Roughly, the key points of our work are described below:

1. We designed a hybrid searchable encryption scheme based on cloud-fog computing. The fog node bridges between the intelligent terminal and the cloud, the data owner and data user can be directly connected to fog nodes, and each fog node is connected to the cloud, reducing unnecessary data transmission.
2. In order to meet the resource-constrained terminal equipment, the novel multi-authority CP-ABE was proposed to support both outsourced encryption and outsourced decryption scheme. Without divulging data privacy, most local calculations are outsourced to fog nodes, enabling data users to enjoy high-rate, low-latency, high-quality services.
3. This article proposes an attribute-based searchable encryption scheme, which realizes one-to-many communication. Data users can query relevant ciphertexts according to the keywords they specify, narrowing the scope of retrieval in massive document.
4. Formal security and performance analysis proves that our scheme is safe and feasible under cloud-fog computing. In addition, it achieve secure data sharing and effectively protect the confidentiality of data.

### 1.3 Organization

The remaining structure of this paper is organized as follows: In Section 2, we review the relevant background knowledge of this scheme. Section 3 presents system model and security model throughout the paper. In Section 4, we give a detailed description of the specific algorithm and the correctness analysis of the scheme. We analyzed the security and discuss the performance of our schemes with comparison to several related works in Section 5 and Section 6. Finally, we conclude this scheme in Section 7.

## 2 Preliminaries

This section mainly gives the basic concept of access structure; then introduces bilinear maps and uses it as the main mathematical tool to construct the encryption algorithm proposed in this paper; the definition of the linear secret sharing scheme is given; and finally some difficult problems are introduced to prove the security of this scheme.

### 2.1 Access structure

In order to achieve fine-grained access control in an ABE scheme, the following access control structure is defined.

**Definition 1 (Access structure** [21]**)**. Let *P* = {*P*_1_, *P*_2_, ⋯, *P*_*n*_} be a set of *n* participants. For ∀*B*,*C*, if B∈A and *B* ⊆ *C*, then C∈A, we call $A\subseteq 2^{\left\{P_{1},\cdots P_{n}\right\}}$ is monotonous. An access structure is a collection A of non-empty subsets of *P* = {*P*_1_, *P*_2_, ⋯, *P*_*n*_}, namely $A\subseteq 2^{\left\{P_{1},\cdots P_{n}\right\}}\backslash \{\emptyset \}$. The sets in A are called the authorized sets, and the sets not in A are called the unauthorized sets.

### 2.2 Bilinear maps

**Definition 2 (Bilinear Maps** [21]**)**. Let G and $G_{T}$ are two groups of prime order *p*. Let *g* be a generator of G. The map $e:G\times G\to G_{T}$ is called a bilinear pairing operation. The mapping *e* satisfies the following properties:

1. Bilinearity: For all u,v∈G and $a,b\in {\mathbb{Z}}_{p}$, we have *e*(*u*^*a*^, *v*^*b*^) = *e*(*u*, *v*)^*ab*^.
2. Non-degenerate: $e(g,g)\neq 1_{G_{T}}$, where $1_{G_{T}}$ is the unit of $G_{T}$.
3. Computability: For all u,v∈G, there is a valid algorithm to calculate *e*(*u*, *v*).

### 2.3 Linear secret sharing scheme

**Definition 3 (Linear Secret Sharing scheme (LSSS)** [6]**)**. Let *P* = {*P*_1_, *P*_2_, ⋯, *P*_*n*_} be a set of participants. (*M*, *ρ*) represents an access structure A, where *M* is the shared generator matrix of *l* × *n* and *ρ* is a mapping. For all *i* = 1, 2, ⋯, *l*, function *ρ* maps the *i* row of *M* to the corresponding attribute. A linear secret sharing scheme consists of the following two effective algorithms:

1. Secret sharing algorithm: To share a secret $s\in {\mathbb{Z}}_{p}$. The algorithm randomly chooses $v_{2},\cdots ,v_{n}\in {\mathbb{Z}}_{p}$ and the column vector **v** = (*s*, *v*_2_, ⋯, *v*_*n*_). Then calculate *λ*_*i*_ = (*M* · **v**)_*i*_, where *λ*_*i*_ belongs to the secret share value obtained by the entity *ρ*(*i*).
2. Secret reconstruction algorithm: Let S∈A be any set of authorized users, we define *I* ⊂ {1, 2, ⋯, *l*} as *I* = {*i*: *ρ*(*i*) ∈ *S*}. There is a constant coefficient ${\left\{\omega_{i}\in {\mathbb{Z}}_{p}\right\}}_{i\in I}$ that satisfies ∑_*i*∈*I*_ *ω*_*i*_*M*_*i*_ = (1, 0, ⋯, 0). The recovered secret will be ∑_*i*∈*I*_ *ω*_*i*_*λ*_*i*_ = *s*. The set of constants can be found in polynomial time.

### 2.4 Hardness assumptions

**Decisional *q*-Parallel Bilinear Diffie-Hellman Exponent (*q*-parallel DBDHE)Assumption** [27] A group G with prime order *p* is selected through security parameters, and *g* is a generator of G. Randomly choose $a,s,b_{1},\cdots ,b_{q}\in {\mathbb{Z}}_{p}$, and given

$$\begin{array}{ll} \text{y}= & (g,g^{s},g^{a},\cdots ,g^{a^{q}},,g^{a^{q+2}},\cdots ,g^{a^{2q}}\\ & \forall_{1\leq j\leq q}\;g^{s\cdot b_{j}},g^{a/b_{j}},\cdots ,g^{a^{q}/b_{j}},,g^{a^{q+2}/b_{j}},\cdots ,g^{a^{2q}/b_{j}}\\ & \forall_{1\leq j,k\leq q,k\neq j}\;g^{a\cdot s\cdot b_{k}/b_{j}},\cdots ,g^{a^{q}\cdot s\cdot b_{k}/b_{j}}) \end{array}$$

It’s hard to distinguish a valid tuple $e{\left(g,g\right)}^{a^{q+1}\cdot s}\in G_{T}$ from a random element *R* in $G_{T}$. An algorithm B outputs υ ∈ {0,1} has advantage *ε* in solving *q*-parallel DBDHE in G if

$$|\text{Pr}[B(\text{y},T=e{\left(g,g\right)}^{a^{q+1}\cdot s})=0]-\text{Pr}[B(\text{y},T=R)=0]|\geq \varepsilon$$

**Definition 4**. we say that the *q*-parallel DBDHE assumption holds if no polynomial time algorithm to solve the *q*-parallel DBDHE problem with non-negligible advantage.

**Decisional Bilinear Diffie-Hellman (DBDH) Assumption** [28,29]. Let *g* be a generator of G and $\beta ,\gamma ,z\in {\mathbb{Z}}_{p}$ be selected at random. If the challenger gives adversary (*g*, *g*^*β*^, *g*^*γ*^, *g*^*z*^), it must be difficult for the adversary to distinguish a valid tuple $e{\left(g,g\right)}^{\beta \gamma z}\in G_{T}$ from a random element $R\in G_{T}$.

An algorithm B outputs υ ∈ {0,1} has advantage *ε* in solving DBDH in G if

$$|\text{Pr}[B(g,g^{\beta },g^{\gamma },g^{z},e{\left(g,g\right)}^{\beta \gamma z})=0]-\text{Pr}[B(g,g^{\beta },g^{\gamma },g^{z},Z=R)=0]|\geq \varepsilon$$

**Definition 5**. we say that the DBDH assumption holds if all polynomial time algorithm have at most a negligible advantage in solving the DBDH problem.

## 3 System overview

### 3.1 Definition of system model

**Fig 2:**
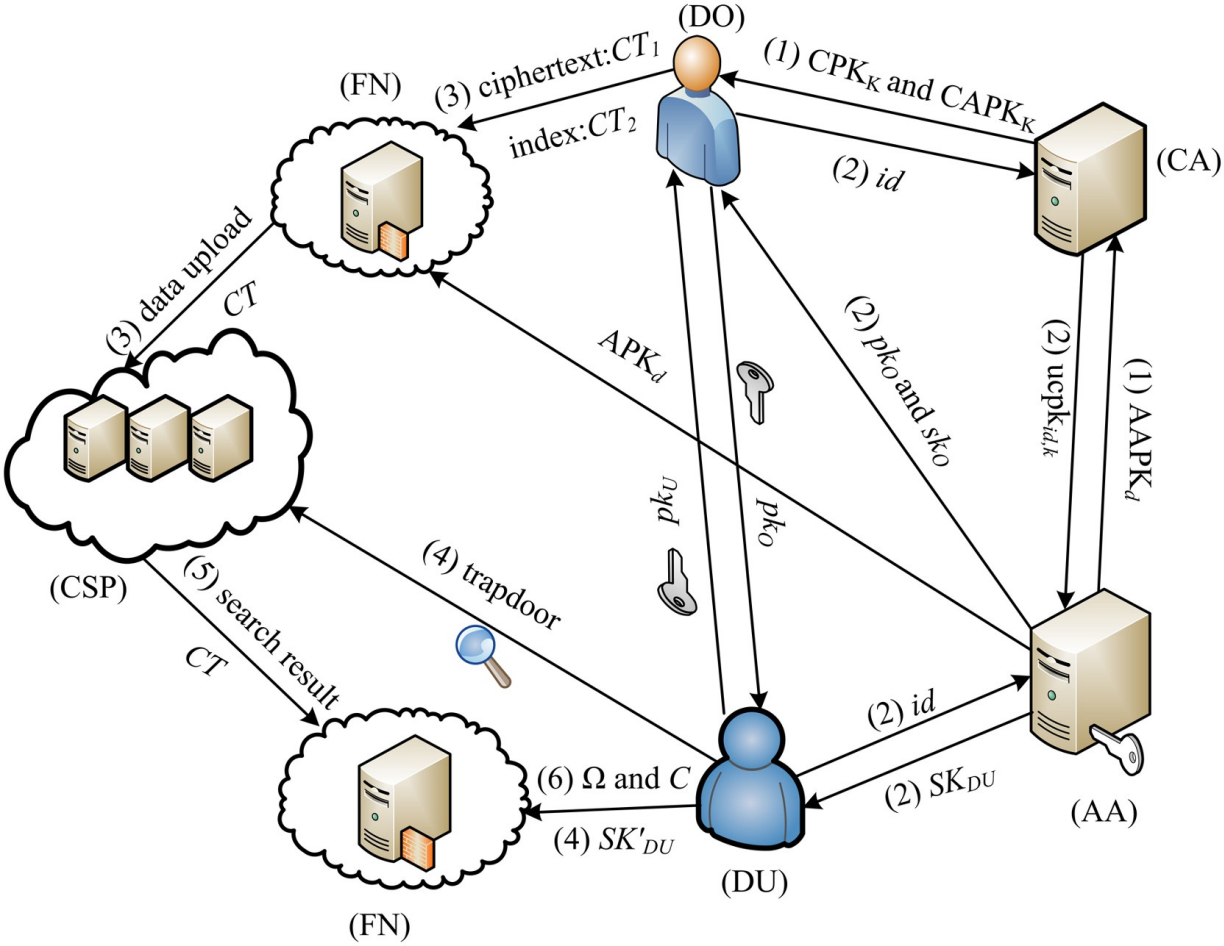
System model.

The system model of this system is shown in Fig 2. The labels (1)-(6) in the figure correspond to the 6 algorithms in our scheme, namely system establishment, key generation, file encryption, trapdoor generation, ciphertext retrieval and file decryption algorithms. It contains the following 6 entities:

**Central Authorities (CA)**: We assumes that there are *K* central authority CA, for example, the health department, education department, state government, etc. The CA generates global public parameters for the PHR system and distribute secret keys based on the user’s globally unique identifier *id*. Each CA works independently and does not need to communicate with each other, and at least one of the CAs is honest and not curious. Note that CA does not participate in any attribute-related operations.

**Attribute Authorities (AA)**: Here are *D* attribute authority AA. Each AA manages different attribute domains *S*_*d*_, $S=\cup_{d=1}^{D}S_{d}$ represents the set of all attributes in the entire system. For ∀*i* ≠ *j* ∈ {1, 2, ⋯, *D*}, where *S*_*i*_ ∩ *S*_*j*_ = ∅. AA generates related secret keys based on the user’s attributes. For example, a hospital can assign the Hospital A attribute to all employees. Health associations can assign different doctors or nurses attributes related to medical professional licenses, such as dermatologists and psychologists. Each AA is independent of each other, one attribute is managed by only one AA, but one AA can manage multiple attributes.

**Cloud Server Provider (CSP)**: The Cloud Server Provider (CSP) is a semi-trusted entity that is mainly responsible for storing encrypted PHR files and keyword indexes, and providing access services for authorized users.

**Fog Nodes (FN)**: A fog node is a trusted entity that is at the edge of the network and has the ability to computing, storage and network services. It is responsible for partial encryption and decryption operations. The fog node helps the DO to generate partial ciphertext and upload all ciphertext to the CSP. It can also decrypt some ciphertext downloaded from the CSP.

**Data Owner (DO)**: The data owner specifies an access policy, encrypts PHR files and keyword sets, and uploads ciphertext and indexes to the CSP.

**Data User (DU)**: The data user downloads the encrypted PHR file from the CSP. The DU can decrypt successfully only if the attributes of the DU satisfy the access policy. For example, doctors and nurses must visit the patient’s PHR file in order to properly diagnose the condition and care.

For a more intuitive description of the scheme, we use personal health records to illustrate. Consider the following scenario: Alice who has a skin disease wants to find an expert to check through an online medical facility. Alice’s needs is (hospital A ∧ dermatologist). In order to protect the confidentiality of personal health records, Alice needs to encrypt medical health records under hospital A ∧ dermatologist condition before uploading data to the CSP. The CSP then searches for a doctor who satisfies hospital A ∧ dermatologist in its own database and sends Alice’s personal health record to the qualified doctor Bob. Bob can decrypt the record for Alice to continue treatment.

### 3.2 Algorithm definition

This scheme consists of 6 algorithms: system setup, key generation, file encryption, trapdoor generation, search over ciphertext, and file decryption. Each algorithm is described as follows:

1. **System Setup**: The algorithm is executed by authority. It contains 3 sub-algorithms:
  **Global—Setup**(1^*λ*^): The global-setup algorithm is run by a trusted third party. It takes as no input other than the security parameters *λ* and outputs the global public parameter GPK.
  **CA—Setup**(GPK, *k*): The CA-setup algorithm is run by each *CA*_*k*_. It input the GPK and the tag *k* of the *CA*, and then output the public parameter (CPK_*k*_, CAPK_*k*_) and the master key CASK_*k*_, and the CAPK_*k*_ is used only by the *CA*_*k*_.
  **AA—Setup**(GPK, *d*, *S*_*d*_): The AA-setup algorithm is performed by each *AA*_*d*_. It takes as inputs the GPK, the tag *d* of the *AA*_*d*_, and attributes set *S*_*d*_ managed by the *AA*_*d*_. It outputs the public parameter (APK_*d*_, AAPK_*d*_) and the master key AASK_*d*_, AAPK_*d*_ is used only by the *AA*_*d*_.
2. **Key Generation**: This algorithm is performed by the *CA*_*k*_ and the *AA*_*d*_. It contains 2 sub-algorithms:
  **CA—KeyGen**(GPK, CMSK_*k*_, AAPK_*d*_, *id*): The CA-key generation algorithm is performed by the *CA*_*k*_. It takes as inputs the global public parameter GPK, the master key CMSK_*k*_ of the *CA*_*k*_, part of the public parameters AAPK_*d*_ of the *AA*_*d*_, and the unique identifier *id* of the data user. It outputs the user-center-key (ucsk_*id*,*k*_, ucpk_*id*,*k*_), where ucpk_*id*,*k*_ is called user-center-public-key.
  $\text{AA-KeyGen}(att,\{{\text{ucpk}}_{id,k}|k\in K\},\{{\text{VerifyKey}}_{k}|k\in K\},{\text{AMSK}}_{d},\text{GPK})$: The AA-key generation algorithm is executed by the *AA*_*d*_. It intakes an attributes *att*, user-center-public-key ucpk_*id*,*k*_, verification key VerifyKey_*k*_, master key AMSK_*d*_, and global public parameters GPK. It outputs the secret key *SK*_*DU*_ of the DU and the public/secret key pair (*pk*_*O*_, *sk*_*O*_) of the DO.
3. **File Encryption**: This algorithm is executed by the FN and the DO. It contains 2 sub-algorithms:
  **Fog—Encrypt**(GPK, {APK_*d*_}, (*M*, *ρ*)): The outsourced encryption algorithm is performed by the FN. The algorithm intakes the global public parameter GPK, the public parameter APK_*d*_ of the *AA*_*d*_, and an access structure (*M*, *ρ*). It outputs the intermediate ciphertext *CT*_Fog_.
  **Do—Encrypt**(*m*, GPK, *CT*_Fog_, *sk*_*O*_, *pk*_*U*_): The local encryption algorithm is usually performed by the DO. It intakes the plaintext message *m*, the global public parameter GPK, DO’s secret key *sk*_*O*_, DU’s public key *pk*_*U*_ and the ciphertext *CT*_Fog_. It outputs all ciphertext *CT*_1_, *CT*_2_ and delivers it to the FN. Finally, the FN uploads the ciphertext *CT* = (*CT*_1_, *CT*_2_) to the CSP.
4. $\text{TrapGen}(\tilde{w},SK_{DU},pk_{O})$: The trapdoor generation algorithm is performed by the DU. It intakes a desired keyword $\bar{w}$, the secret key *SK*_DU_ and the public key *pk*_*O*_. Finally, it outputs the trapdoor $T_{\tilde{w}}$ and the pre-decryption key $SK_{DU}^{\prime }$.
5. **Search over Ciphertext**: This algorithm is executed by the CSP to determine whether the keywords in the ciphertext match the keywords of the trapdoor.
  $\text{Search}(CT_{2},T_{\tilde{w}},pk_{O},pk_{U})$: The search algorithm is performed by the CSP. It intakes the index *CT*_2_, trapdoor $T_{\tilde{w}}$ and the public key *pk*_*O*_, *pk*_*U*_. If the trapdoor and index match successfully, returns the search result to the FN.
6. **File Decryption**: This algorithm is executed by the FN and the DU. Including 2 sub-algorithms:
  $\text{Fog-Decrypt}(CT,SK_{\text{DU}}^{\prime })$: The outsourced decryption algorithm is performed by the FN. It intakes the ciphertext *CT* and the pre-decryption key $SK_{\text{DU}}^{\prime }$. It outputs the partially decrypted ciphertext (*C*, Ω) to the DU.
  **Do—Decrypt**(*C*, Ω, *RK*): The final decryption algorithm is performed by the DU. It intakes the partially decrypted ciphertext (*C*, Ω) and the retrieval key *RK*. It outputs the plaintext message *m*.

### 3.3 Definition of security model

In the fog-cloud storage system, the CSP is also curious about the contents of the encrypted data. We assume that the CSP will correctly perform the tasks assigned by the central authority and attribute authority. The AA and CA can be corrupted or attacked. To demonstrate the security of our scheme, we design two security games: indistinguishability against selective ciphertext-policy and chosen ciphertext attack (IND-sCP-CCA) game and trapdoor privacy game.

#### Game 1. Ciphertext indistinguishability

The security of this scheme is defined by the following game run between a challenger B and an adversary A. A can corrupt CAs and AAs by specifying $K_{c}^{\prime }\subset K$ and $D_{c}^{\prime }\subset D$ after seeing the public parameters, where $K\backslash K_{c}^{\prime }\neq \emptyset$ and $D\backslash D_{c}^{\prime }\neq \emptyset$. The security game is defined as follows:

**Init**. A exposes a challenged access structure (M*, ρ*), where M* is an l* × n* ≤ *q* matrix.

**Setup**. B runs algorithms **Global—Setup**, **CA—Setup** and **AA—Setup**. The public parameter GPK, (CPK_*k*_, CAPK_*k*_) and (APK_*d*_, AAPK_*d*_) are sent to A. We allow A to corrupt authority $K_{c}^{\prime }\subset K$ and $D_{c}^{\prime }\subset D$(where $K\backslash K_{c}^{\prime }\neq \emptyset$, $D\backslash D_{c}^{\prime }\neq \emptyset$). A submits an access policy (M*, ρ*) to B, where M* is a matrix of l* × n* ≤ *q*, ρ* maps the rows of M* to attributes. For uncorrupted authorities in $K\backslash K_{c}^{\prime }$ and $D\backslash D_{c}^{\prime }$, B sends only the public keys to A. For corrupted authorities $K_{c}^{\prime }$ and $D_{c}^{\prime }$, B sends both the master key $\left\{{\text{CMSK}}_{k}|k\in K_{c}^{\prime }\right\}$ and $\left\{{\text{AMSK}}_{d}|d\in D_{c}^{\prime }\right\}$ to A.

**Phase 1**. A can performs the following secret key queries many times, in which limiting the secret key attribute set to be queried does not meet the access policy M* to be challenged. In other words, A cannot ask for a key which can be decrypt in combination with any keys that can obtained from corrupted CA_S_ and AA_S_:

**CKQ**(*id*, *k*): For each unpurchased $CA_{k}(k\in K\backslash K_{c}^{\prime })$, A submits a tuple (*id*, *k*) to B, where *id* is the user’s global identifier and *k* is the tag of an uncorrupted authority *CA*. B runs the algorithm **CA—KeyGen** and returns the corresponding user-central-key (ucsk_*id*,*k*_, ucpk_*id*,*k*_) to A.

$\text{AKQ}(att,\{{\text{ucpk}}_{id,k}|k\in K\},d)$: For each unpurchased $AA_{d}(d\in D\backslash D_{c}^{\prime })$, A submits a tuple $\left(att,\{{\text{ucpk}}_{id,k}|k\in K\},d\right)$ to B, where *att* is an attribute of the attribute set $S_{d},\;\{{\text{ucpk}}_{id,k}|k\in K\}$ are the central-public-keys of the user *id*, and *d* is the tag of an uncorrupted authority *AA*. B runs algorithm **AA—KeyGen** and outputs ⊥ if ucpk_*id*,*k*_ are invalid. Otherwise, user-attribute-key uask_*att*,*id*_ are returned to A.

**Challenge**. A sends two equal length messages *m*_0_ and *m*_1_. B selects a random bit *υ* ∈ {0, 1}, and encrypts *m*_*υ*_ under (M*, ρ*). Then return the challenge ciphertext $CT_{1}^{*}$ to A.

**Phase 2**. A conducts more secret key queries similar to **Phase 1**.

**Guess**. A submits a guess *υ*′ ∈ {0, 1}. If *υ*′ = *υ*, the A wins the safety game, otherwise A fails.

The advantage of the A in breaking this game is $Adv_{A}^{\text{IND-sCP-CCA}}=|\text{Pr}[\upsilon =\upsilon^{\prime }]-\frac{1}{2}|$.

**Definition 6**. The proposed scheme is IND-sCP-CCA secure if all polynomial time adversary A have at most a negligible advantage in the above security game.

#### Game 2. Trapdoor privacy

**Setup**. Given a security parameter *λ*, B generates the DU’s public key *pk*_*U*_ and the DO’s public key *pk*_*O*_.

**Phase 1**. A adaptively issues polynomial many times following queries.

**Trapdoor Querie** $O_{T}$: A can ask any keyword’s trapdoor.

**Index Queries** $O_{I}$: A can ask any keyword’s index.

**Challenge**. A sends two equal length keywords $\left(w_{0}^{*},w_{1}^{*}\right)$, with the restriction that $\left(w_{0}^{*},w_{1}^{*}\right)$ have not been queried for trapdoors nor indexes. B selects a random bit *υ* ∈ {0, 1} and generates the trapdoor $T_{w_{\upsilon }^{*}}$ of keyword $w_{b}^{*}$ and returns it to A.

**Phase 2**. Same as **Phase 1**, with the restriction $w\neq w_{0}^{*},w_{1}^{*}$.

**Guess**. A submits a guess *υ*′ ∈ {0, 1}. If *υ*′ = *υ*, the A wins this game, otherwise A fails.

The advantage of the A in breaking this game is $Adv_{A}=|\text{Pr}[\upsilon =\upsilon^{\prime }]-\frac{1}{2}|$.

**Definition 7**. The proposed scheme is trapdoor privacy secure if all polynomial time adversary A have at most a negligible advantage in the above security game.

## 4 Algorithm construction

In this part, the scheme address the hospital scene to construct a PHR sharing scheme based on cloud-fog computing. The patient encrypts personal medical data according to different access policies and stores it in the cloud. Doctors need to download the PHR file from the cloud if they want to view the case. As the distance of transmission of encrypted data from the cloud to the mobile device is going up, communication costs and delays are increasing. By deploying the fog server in the hospital, the total response time is reduced. In hospitals, it is difficult for a dermatologist to obtain dermatological data in a massive medical database. Using keyword-based search technology can not only access the data in the cloud, but also perform keyword search directly in the cloud. The doctor only downloads the files he needs, effectively reducing communication costs.

### 4.1 Detailed description of our scheme

#### A. System setup

During the system setup phase, global public parameters are generated. The *CA*_*k*_ and *AA*_*d*_ generate their own public key and secret key, respectively. The phase contains 3 sub-algorithms: **Global—Setup**, **CA—Setup** and **AA—Setup**.

**Global—Setup**(1^*λ*^): Only trusted third parties run the algorithm. It takes as no input other than the security parameters *λ*. This algorithm chooses a bilinear map $e:G\times G\to G_{T}$, where G and $G_{T}$ are two multiplicative cyclic groups of prime order *p* (*p* > 2^*λ*^). Let *g* be a generator of G, and randomly pick h∈G. The Σ_*sign*_ = (KeyGen, SignKey, VerifyKey) is a secure unforgeable signature scheme. It also chooses a hash function $H:{\left\{0,1\right\}}^{*}\to G$. Return the global public parameters $\text{GPK}=(G,G_{T},p,g,h,\sum_{sign},H_{1})$.

**CA—Setup**(GPK, *k*): Each *CA*_*k*_ runs this algorithm. It takes as inputs the global public parameters GPK and the tag *k* of the *CA*. *CA*_*k*_ runs the key generation algorithm **KeyGen** → (SignKey_*k*_, VerifyKey_*k*_) of the scheme Σ_*sign*_ and selects a random exponent $\alpha_{k}\in {\mathbb{Z}}_{p}$. Then Central authority publishes the public key ${\text{CPK}}_{k}=e{\left(g,g\right)}^{\alpha_{k}}$, CAPK_*k*_ = VerifyKey_*k*_ and keeps the master key CMSK_*k*_ = (*α*_*k*_, SignKey_*k*_) secret.

**AA—Setup**(GPK, *d*, *S*_*d*_): Each *AA*_*d*_ runs this algorithm. It takes as inputs the global public parameter GPK, the tag *d* of the *AA* and *S*_*d*_ is a set of attributes managed by *AA*_*d*_. For each attribute *att* ∈ *S*_*d*_, select $s_{att}\in {\mathbb{Z}}_{p}$ and calculates $T_{att}=g^{s_{att}}$. For each *k* ∈ *K*, *AA*_*d*_ randomly chooses $v_{d,k}\in {\mathbb{Z}}_{p}$ and computes $V_{d,k}=g^{v_{d,k}}$. Then it publishes APK_*d*_ = {*T*_*att*_ | *att* ∈ *S*_*d*_}, AAPK_*d*_ = {*V*_*d*,*k*_ | *k* ∈ *K*} as its public key and the master key is kept as AMSK_*d*_ = ({*s*_*att*_ | *att* ∈ *S*_*d*_}, {*V*_*d*,*k*_ | *k* ∈ *K*}).

#### B. Key generation

**Fig 3:**
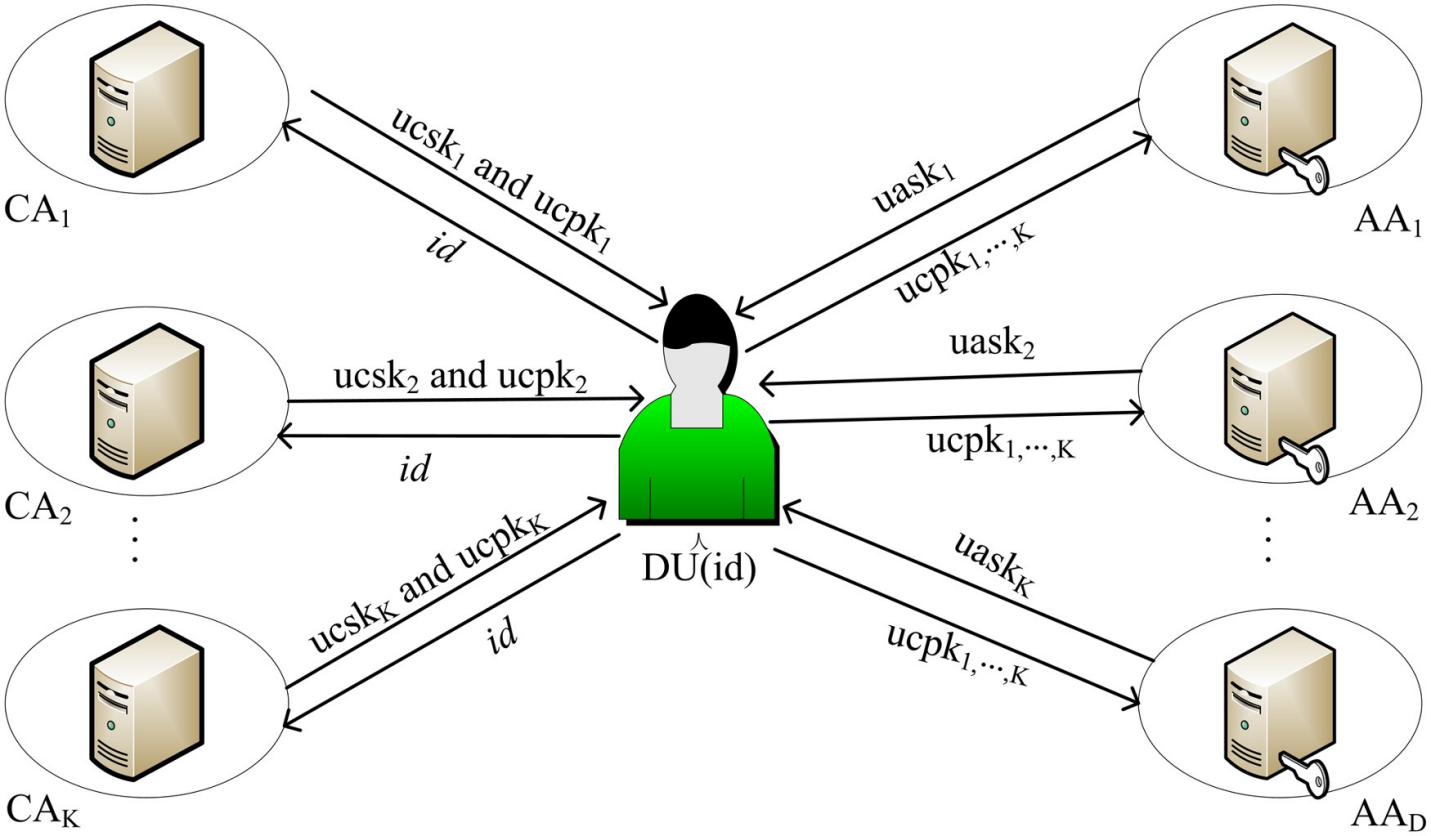
CA_S_ and AA_S_ send key for user *id*.

We assume that there are two central authorities: the health department, the education department, and two attribute authorities: hospitals, health associations. Because the keys and attribute sets are related in the CP-ABE scheme, the attribute authority *AA*_*d*_ will generate a corresponding attribute key according to the user’s attribute set. Consider the following scenario: Generate a key for a dermatologist working in Hospital A. The key generation phase contains 2 sub-algorithms: **CA—KeyGen** and **AA—KeyGen**. The key generation process is shown in Fig 3.

**CA—KeyGen**(GPK, CMSK_*k*_, AAPK_*d*_, *id*): A data user sends its globally unique identifier *id* to the *CA*_*k*_ to request the user-central-key. The *CA*_*k*_ randomly picks element $r_{id,k}\in {\mathbb{Z}}_{p}$ and sets the user-center-key: ${\text{ucsk}}_{id,k}=g^{\alpha_{k}}h^{r_{id,k}}$, $\Gamma_{id,k}=g^{r_{id,k}}$. For *d* ∈ [1, *D*], *CA*_*k*_ calculates $L_{id,k,d}=V_{d,k}^{r_{id,k}}$ and generates the signature *sign*_*id*,*k*_ = Sign(SignKey_*k*_, *id*‖*k*‖Γ_*id*,*k*_‖{*L*_*id*,*k*,*d*_}_*d*∈[1,*D*]_). Simultaneously, the user-central-public-key ${\text{ucpk}}_{id,k}=(id,k,\Gamma_{id,k},\{L_{id,k,d}|d\in D\},\delta_{id,k})$ is output.

$\text{AA-KeyGen}(att,\{{\text{ucpk}}_{id,k}|k\in K\},\{{\text{VerifyKey}}_{k}|k\in K\},{\text{AMSK}}_{d},\text{GPK})$: For attribute *att* ∈ *S*_*d*_. The data user *id* sends its user-center-public-key $\left\{{\text{ucpk}}_{id,k}|k\in K\right\}$ to the *AA*_*d*_ to request the user-attribute-key.

① For any *k* ∈ [1, *K*], the *AA*_*d*_ verifies the following equation:

$$\text{Verify}({\text{VerifyKey}}_{k},id∥k∥\Gamma_{id,k}∥{\left\{L_{id,k,d}\right\}}_{d\in [1,D]}∥sign_{id,k})\overset{?}{\to }True$$

$$e(g,L_{id,k,d})=e(V_{d,k},\Gamma_{id,k})$$

If the above equation holds, then ② is performed. Otherwise, the *AA*_*d*_ outputs ⊥. It indicates that the user-central-public-key ucpk_*id*,*k*_ submitted by user is invalid.

② For any *k* ∈ [1, *K*], the *AA*_*d*_ generates the user-attribute-key uask_*att*,*id*_ for the user

$${\text{uask}}_{att,id}=\prod_{k=1}^{K}{\left(L_{id,k,d}\right)}^{s_{att}/v_{d,k}}\;=\prod_{k=1}^{K}{\left(g^{v_{d,k}\cdot r_{id,k}}\right)}^{s_{att}/v_{d,k}}\;=T_{att}{}^{\sum_{k=1}^{K}r_{id,k}}$$

After that, algorithm randomly picks $\beta ,\gamma \in {\mathbb{Z}}_{p}^{*}$ and computes *g*^*β*^, *g*^*γ*^. It returns the public/secret key pair of DO’s and DU’s as (*pk*_*O*_, *sk*_*O*_) = (*g*^*γ*^, *γ*) and (*pk*_*U*_ = *g*^*β*^, *sk*_*U*_ = *β*), respectively. So DU’s secret key *SK*_DU_ = ({ucsk_*id*,*k*_, Γ_*id*,*k*_ | *k* ∈ *K*}, {uask_*att*,*id*_ | *att* ∈ *S*_*d*_}, *β*).

#### C. File encryption

Before uploading the PHR file to the CSP, the patient needs to encrypt the file based on the access policy (hospital A ∧ dermatologist) and sends an access policy to FN. The encryption algorithm contains two sub-algorithms: **Fog—Encrypt** and **Do—Encrypt**.

**Fog—Encrypt**(GPK, {APK_*d*_}, (*M*, *ρ*)): Here (*M*, *ρ*) is an LSSS access structure. Assuming that *M* is an *l* × *n* matrix. Function *ρ*(.), which is an injective function, maps each rows of *M* to different attributes. For ∀*i* ∈ {1, 2, ⋯, *l*}, fog nodes are randomly selects $\lambda_{i}{}^{\prime },r_{i}\in {\mathbb{Z}}_{p}^{*}$ and sets the ciphertext *CT*_*Fog*_ as follows:

$$C_{i,1}=h^{\lambda_{i}{}^{\prime }}\cdot T_{\rho (i)}^{-r_{i}},\;C_{i,2}=g^{r_{i}}$$

Output part of the ciphertext $CT_{\text{Fog}}=((M,\rho ),{\left\{C_{i,1},C_{i,2},\lambda_{i}^{\prime }\right\}}_{i\in [1,l]})$.

**Do—Encrypt**(*m*, GPK, *CT*_Fog_, *sk*_*O*_, *pk*_*U*_): *m* is the PHR file to be shared by patient. The DO first creates vectors $\text{V}=(s,v_{2},\cdots ,v_{n})\in {\mathbb{Z}}_{p}^{n}$, where *s* is the random secret to be shared. From *i* = 1 to *l*, it gets the sub-secret *λ*_*i*_ = **V** · ***M***_*i*_ by computing, where *M*_*i*_ is the *i* − th row of matrix *M*. The intact data ciphertext *CT*_1_ can be created by the following calculation:

$$C=m\cdot \overset{K}{\underset{k=1}{\prod }}e{\left(g,g\right)}^{\alpha_{k}\cdot s},\;C^{\prime }=g^{s},\;\{C_{i,3}=\lambda_{i}-\lambda_{i}{}^{\prime }|i\in [1,l]\}$$

All data ciphertext is published as *CT*_1_ = {(*M*, *ρ*), *C*, *C*′, {*C*_*i*,1_, *C*_*i*,2_, *C*_*i*,3_}_*i*∈[1,*l*]_}.

Then, the DO extracts keywords from the PHR file to form a set of keywords *W*_*m*_. For each keyword *w*_*i*_ ∈ *W*_*m*_, the algorithm randomly selects $t_{i}\in {\mathbb{Z}}_{p}^{*}$ to generate a keyword index $CT_{2}=({\left\{I_{i}\right\}}_{w_{i}\in W_{m}})$ and send it to the FN.

$$I_{1,i}=H{\left(w_{i}\right)}^{sk_{o}}\cdot g^{t_{i}},\;I_{2,i}=pk_{U}{}^{t_{i}}$$

Subsequently, the FN uploads the ciphertext *CT* = (*CT*_1_, *CT*_2_) to the CSP.

#### D. Trapdoor generation

After the doctor issues a search request to the CSP, it generates trapdoors to search for keywords. The trapdoor generation process as follows:

$\text{TrapGen}(\tilde{w},SK_{DU},pk_{O})$: It takes search query for the keyword $\tilde{w}$ as inputs. It creates the trapdoor as $T_{\tilde{w}}=e(H{\left(\tilde{w}\right)}^{sk_{U}},pk_{O})$. The DU delivers the trapdoor $T_{\tilde{w}}$ to the CSP. Then DU chooses a random number $\delta \in {\mathbb{Z}}_{p}^{*}$ to blind his secret key and computes $\text{ucs}{\text{k}}_{id,k}^{\prime }={\left(g^{\alpha_{k}}h^{r_{id,k}}\right)}^{\delta }$, $\Gamma_{id,k}^{\prime }={\left(g^{r_{id,k}}\right)}^{\delta }$, ${\text{uask}}_{att,id}^{\prime }={\left(T_{att}{}^{\sum_{k=1}^{K}r_{id,k}}\right)}^{\delta }$. DU holds the unique retrieval key *RK* = *δ*. Finally, the DU outputs the pre-decryption key $SK_{\text{DU}}^{\prime }=(\{\text{ucs}{\text{k}}_{id,k}^{\prime },\Gamma_{id,k}^{\prime }|k\in K\},\{\text{uas}{\text{k}}_{att,id}^{\prime }|att\in S_{d}\})$ and sends it to the FN.

#### E. Search over ciphertext

When the CSP receives the user’s search request, the CSP performs the following algorithm to search the matched health records:

$\text{Search}(CT,T_{\tilde{w}},pk_{O},pk_{U})$: When gained the DU’s search query, the CSP first checks whether the DU’s attribute set satisfies with access structure (*M*, *ρ*). If it is true, the CSP uses the following equation to checks if the trapdoor $T_{\tilde{w}}$ and index *CT*_2_ match.

$$e(I_{1,i},pk_{U})=T_{\tilde{w}}\cdot e(I_{2,i},pk_{O})$$

If the equation does not hold, ⊥ is returned. Otherwise, it indicates that the matching is successful and then the CSP returns the corresponding search results *CT* to the FN.

#### F. File decryption

If the doctor’s attribute satisfies the access policy, the doctor uses retrieval key to successfully decrypt the partially encrypted ciphertext and obtain the patient’s PHR file. The decryption algorithm includes 2 aspects: **Fog—Decrypt** and **Do—Decrypt**.

$\text{Fog-Decrypt}(CT,SK_{\text{DU}}^{\prime })$: The FN downloads the ciphertext *CT* from the CSP and the pre-decryption key $SK_{\text{DU}}^{\prime }$ from the DU. Once that attribute set of the DU does not satisfy the access policy, the FN outputs ⊥. If not, there must be a constant set ${\left\{c_{i}\in {\mathbb{Z}}_{p}\right\}}_{i\in I}$ that satisfies ∑_*i*∈*I*_*c*_*i*_*M*_*i*_ = (1, 0, ⋯, 0). If {*λ*_*i*_} is a valid share of secret *s*, there is ∑_*i*∈*I*_*c*_*i*_*λ*_*i*_ = *s*. The FN uses the pre-decryption key $SK_{\text{DU}}^{\prime }$ to calculate the intermediate ciphertext Ω as follows:

$$\Omega ==\frac{\prod_{k=1}^{K}e(C^{\prime },\text{ucs}{\text{k}}_{id,k}^{\prime })}{\prod_{i\in I}{\left(e(C_{i,1}\cdot h^{C_{i,3}},\prod_{k=1}^{K}\Gamma_{id,k}^{\prime })e(C_{i,2},\text{uas}{\text{k}}_{att,id}^{\prime })\right)}^{c_{i}}}=e{\left(g,g\right)}^{s\delta \sum_{k=1}^{K}\alpha_{k}}\;\text{where}\;r_{id}=\overset{K}{\underset{k=1}{\sum }}r_{id,k}$$

Finally, the FN sends the partially decryption result (*C*, Ω) to the DU.

**Du—Decrypt**(*C*, Ω, *RK*): After receiving the partially decrypted ciphertext (*C*, Ω) by the DU, the DU decrypts it with retrieval key *RK* = *δ*. The plaintext *m* can be recovered by the following equation.

$$m=\frac{C}{\Omega }=\frac{m\cdot \prod_{k=1}^{K}e{\left(g,g\right)}^{\alpha_{k}\cdot s}}{{\left(e{\left(g,g\right)}^{s\delta \sum_{k=1}^{K}\alpha_{k}}\right)}^{\frac{1}{\delta }}}\;\text{where}\;\alpha =\overset{K}{\underset{k=1}{\sum }}\alpha_{k}$$

### 4.2 Correctness analysis

In this part, we will prove the correctness of our scheme by the following equations:

(1) In order to verify whether the user-central-public-key ucpk_*id*,*k*_ is valid, the calculation process is as follows:
  $$e(g,L_{id,k,d})=e(g,V_{d,k}^{r_{id,k}})=e{\left(g,g\right)}^{v_{d,k}r_{id,k}}$$
  $$e(V_{d,k},\Gamma_{id,k})=e(g^{v_{d,k}},g^{r_{id,k}})=e{\left(g,g\right)}^{v_{d,k}r_{id,k}}$$
(3) The index and trapdoor matching process is verified as follows:
  $$\begin{array}{ll} e(I_{1,i},pk_{U}) & =e(H{\left(w_{i}\right)}^{\gamma }\cdot g^{t_{i}},g^{\beta })\\ & =e(H{\left(w_{i}\right)}^{\gamma },g^{\beta })\cdot e(g^{t_{i}},g^{\beta }) \end{array}$$
  $$T_{\tilde{w}}\cdot e(I_{2,i},g)=e(H{\left(\tilde{w}\right)}^{\beta },g^{\gamma })\cdot e(g^{\beta t_{i}},g)$$
(4) The Fog-Decryption process calculates:
  $$\begin{array}{ll} \Omega & =\frac{\prod_{k=1}^{K}e(C^{\prime },\text{ucs}{\text{k}}_{id,k}^{\prime })}{\prod_{i\in I}{\left(e(C_{i,1}\cdot h^{C_{i,3}},\prod_{k=1}^{K}\Gamma_{id,k}^{\prime })e(C_{i,2},\text{uas}{\text{k}}_{att,id}^{\prime })\right)}^{c_{i}}}\\ & =\frac{\prod_{k=1}^{K}e(g^{s},{\left(g^{\alpha_{k}}h^{r_{id,k}}\right)}^{\delta })}{\prod_{i\in I}{\left(e(h^{\lambda_{i}}\cdot g^{-s_{att}r_{i}},g^{\delta r_{id}})e(g^{r_{i}},{\left(g^{s_{att}\cdot \sum_{k=1}^{K}r_{id,k}}\right)}^{\delta })\right)}^{c_{i}}}\\ & =\frac{e{\left(g,g\right)}^{s\delta \sum_{k=1}^{K}\alpha_{k}}e(g^{s},h^{\delta r_{id}})}{e{\left(h,g^{\delta r_{id}}\right)}^{\sum_{i\in I}\lambda_{i}c_{i}}}\\ & =e{\left(g,g\right)}^{s\delta \sum_{k=1}^{K}\alpha_{k}} \end{array}$$

## 5 Security analysis

**Theorem 1**. Our system is secure against IND-sCP-CCA based on the standard model if the decisional *q*—parallelBDHE assumption holds and the signature scheme ∑_*sign*_ is existent and unforgeable.

**Proof**. Assuming that exists a polynomial time adversary A who can break the IND-sCP-CCA security of our construction with the advantage of $\varepsilon =Adv_{A}^{\text{IND-sCP-CCA}}$. In the following security game, given the decisional *q*—parallelBDHE problem instance $\left(p,G,G_{T},\vec{y},T\right)$, we can build a simulator B to decide whether $T=e{\left(g,g\right)}^{a^{q+1}\cdot s}$ or not. A can ask for the master key of any buying center authority $K_{c}^{\prime }$ and attribute authority $D_{c}^{\prime }$. The interaction between B and A is as follows:

**Init**. B is given a decisional *q*—parallelBDHE problem instance $\left(p,G,G_{T},\vec{y},T\right)$. A exposes the access structure (M*, ρ*) to be challenged, where M* is a l* × n* ≤ *q* matrix.

**Setup**. To expose public parameters, B performs the following operations:

1. B selects g,h∈G, sets *h* = *g*^*a*^. B sets global public parameter GPK and sends it to A.
2. For each non-purchased $CA_{k}(k\in K\backslash K_{c}^{\prime })$, B randomly chooses ${\hat{\alpha }}_{k}\in {\mathbb{Z}}_{p}$ and implicitly sets $\alpha_{k}={\hat{\alpha }}_{k}+a^{q+1}$ by letting ${\text{CPK}}_{k}=e(g^{a},g^{a^{q}})\cdot e{\left(g,g\right)}^{{\hat{\alpha }}_{k}}$. B selects the unforgeable signature algorithm ∑_*sign*_ and calls the algorithm **KeyGen** → (SignKey_*k*_, VerifyKey_*k*_) to generate a signature key pair, and sets CAPK_*k*_ = VerifyKey_*k*_.
3. Symbol *X* denote a set which indices *i* satisfied ρ*(*i*) = *x*. It means that all the row in the set *X* match the same attribute *x*. For any *x*(1 ≤ *x* ≤ *S*), choose the random exponent ${\hat{u}}_{x},v_{d,k}\in {\mathbb{Z}}_{p}$. B calculates:
  $${\text{APK}}_{d}=T_{x}=g^{{\hat{u}}_{x}}\cdot \prod_{i\in X}g^{\frac{aM_{i,1}^{*}}{b_{i}}+\frac{a^{2}M_{i,2}^{*}}{b_{i}}+\cdots +\frac{a^{n^{*}}M_{i,n^{*}}^{*}}{b_{i}}}$$
  $${\text{AAPK}}_{d}=V_{d,k}=g^{v_{d,k}}$$

Finally, B sends GPK, $\left(\{{\text{CPK}}_{k},{\text{CAPK}}_{k}\}|k\in K\right)$ and $\left(\{{\text{APK}}_{d},{\text{AAPK}}_{d}\}|d\in D\right)$ to A. Note that the simulated public parameters have the same distribution as the actual parameters.

**Phase1**. At this stage, B accepts secret key queries from the A. Limiting B receives a secret key query for a set *S* which does not satisfy M*.

**CKQ**(*id*, *k*): For each non-purchased $CA_{k}(k\in K\backslash K_{c}^{\prime })$, A submits a tuple (*id*, *k*) to B. According the definition of LSSS, it is not hard to find that there exist a vector $\text{w}=(w_{1,k},w_{2,k},\cdots ,w_{n^{*},k})\in {\mathbb{Z}}_{p}^{n^{*}}$ such that *w*_1,*k*_ = −1. For any *i* where ρ*(*i*) ∈ *S*, we have that $\sum_{j=1}^{n^{*}}w_{j,k}\cdot M_{i,j}^{n^{*}}=0$. B chooses a random number $r_{id,k}^{\prime }\in {\mathbb{Z}}_{p}$, then implicitly define *r*_*id*,*k*_ as

$$r_{id,k}=r_{id,k}^{\prime }+w_{1,k}a^{q}+w_{2,k}a^{q-1}+\cdots +w_{n^{*},k}a^{q-n^{*}+1}$$

It performs this by setting

$$\Gamma_{id,k}=g^{r_{id,k}^{\prime }+w_{1,k}a^{q}+\cdots +w_{n^{*},k}a^{q-n^{*}+1}}=g^{r_{id,k}^{\prime }+\sum_{i\in [n^{*}]}w_{i,k}a^{q-i+1}}=g^{r_{id,k}}$$

The parameter $g^{a^{q+1}}$ is included during calculating ucsk_*id*,*k*_. By defining *r*_*id*,*k*_, we find that $g^{ar_{id,k}}$ contains a term $g^{-a^{q+1}}$. Therefore, the parameter $g^{a^{q+1}}$ included in ucsk_*id*,*k*_ can be eliminated. B calculates ucsk_*id*,*k*_, *L*_*id*,*k*,*d*_ as follows:

$$\begin{array}{ll} {\text{ucsk}}_{id,k} & =g^{{\hat{\alpha }}_{k}}g^{ar_{id,k}^{\prime }}\cdot \prod_{i=2}^{n^{*}}g^{a^{q+2-i}\cdot w_{i.k}}\\ & =g^{{\hat{\alpha }}_{k}}g^{ar_{id,k}^{\prime }}g^{a^{q+1}}g^{-a^{q+1}}g^{a^{q}w_{2,k}}\cdots g^{a^{q+2-n^{*}}\cdot w_{i.k}}\\ & =g^{\alpha_{k}}h^{r_{id,k}^{\prime }+w_{1,k}a^{q}+\cdots +w_{i.k}a^{q+1-n^{*}}} \end{array}$$

$$L_{id,k,d}=\Gamma_{id,k}^{v_{d,k}}=g^{r_{id,k}\cdot v_{d,k}}$$

B generates a signature *sign*_*id*,*k*_ and returns ucsk_*id*,*k*_, ${\text{ucpk}}_{id,k}=(id,k,\Gamma_{id,k},\{L_{id,k,d}|d\in D\},sign_{id,k})$ gives A.

$\text{AKQ}(att,\{{\text{ucpk}}_{id,k}|k\in K\},d)$: For each non-purchased $AA_{d}(d\in D\backslash D_{c}^{\prime })$, A submits a tuple $\left(att,\{{\text{ucpk}}_{id,k}|k\in K\},d\right)$ to the B. Then B calls algorithm **AA—KeyGen** to verify the validity of the signature on the ucpk_*id*,*k*_. If the validation is successful, B calculates ucsk_*att*,*id*_ as follow, otherwise output ⊥.

$$\begin{array}{ll} {\text{uask}}_{att,id} & =T_{att}^{{\hat{u}}_{att}}\cdot \prod_{k=1}^{K}\prod_{i\in X}\prod_{j=1}^{n^{*}}{\left(g^{(\frac{a^{j}}{b_{i}})\cdot r_{id,k}^{\prime }}\cdot \prod_{y=1,y\neq j}^{n^{*}}g^{(\frac{a^{q+1+j-y}}{b_{i}})\cdot w_{y,k}}\right)}^{M_{i,j}^{*}}\\ & =T_{att}^{{\hat{u}}_{att}}\cdot \prod_{k=1}^{K}g^{(\sum_{i\in X}\sum_{j=1}^{n^{*}}(\frac{a^{j}}{b_{i}})\cdot M_{i.j}^{*})r_{id,k}^{\prime }}g^{\left(\sum_{i\in X}\sum_{j=1}^{n^{*}}\sum_{y=1,y\neq j}^{n^{*}}(\frac{a^{q+1+j-y}}{b_{i}})\cdot w_{y,k}\cdot M_{i.j}^{*}\right)}\\ & =\prod_{k=1}^{K}g^{\left(\sum_{i\in X}\frac{aM_{i,1}^{*}}{b_{i}}+\frac{a^{2}M_{i,2}^{*}}{b_{i}}+\cdots +\frac{a^{n^{*}}M_{i,n^{*}}^{*}}{b_{i}}\text{+}{\hat{u}}_{att})(r_{id,k}^{\prime }+\sum_{y=1}^{n^{*}}w_{y,k}\cdot a^{q+1-y}\right)}\\ & =g^{\sum_{k=1}^{K}r_{id,k}s_{att}} \end{array}$$

Finally, B returns *SK*_DU_ = ({ucsk_*id*,*k*_, Γ_*id*,*k*_ | *k* ∈ *K*}, {uask_*att*,*id*_ | *att* ∈*S*_*d*_}) to the A.

**Challenge**. After A finished the query **Phase1**, he sends two equal length messages *m*_0_ and *m*_1_ to B. B then throws a random bit υ ∈ {0, 1} and encrypts *m*_*υ*_. It creates challenge ciphertext

$$C=m_{\upsilon }\cdot T\cdot \prod_{k=1}^{K}e(g^{{\hat{\alpha }}_{k}},g^{s})$$

$$C^{\prime }=g^{s}$$

The most difficult things for B is to simulate *C*_*i*,1_ since it contains terms $g^{a^{j}s}$. However, B can do the secret splitting, so that these items can be cancel out. B chooses random and implicitly sets vector $\text{V}=(s,sa+\chi_{2},sa^{2}+\chi_{3},\cdots ,sa^{n^{*}-1}+\chi_{n^{*}})\in {\mathbb{Z}}_{p}^{n^{*}}$ to share secret *s*. For vector **V**, the sharing of the secret *s* can be constructed as $\begin{array}{ll} \lambda_{i} & =(\text{v},M_{i}^{*})\\ & =s\cdot M_{i,1}^{*}+(sa+\chi_{2})M_{i,2}^{*}+(sa^{2}+\chi_{3})M_{i,3}^{*}+\cdots +(sa^{n^{*}-1}+\chi_{n^{*}})M_{i,n^{*}}^{*}\\ & =s\cdot M_{i,1}^{*}+\sum_{j=2}^{n^{*}}(sa^{j-1}+\chi_{j})\cdot M_{i,j}^{*} \end{array}$.

Let *R*_*i*_ be a collection of *i* ≠ *y*(*i* ∈ [1, l*]), such that ρ*(*i*) = ρ*(*y*). Intuitively, B randomly chooses $\lambda_{i}^{\prime \prime },{\hat{r}}_{1},\cdots ,{\hat{r}}_{l^{*}}\in {\mathbb{Z}}_{p}^{*}$. For each row of matrix M*, set ρ*(*i*) = x*. By implicitly setting $r_{i}=-{\hat{r}}_{i}-sb_{i}$. We can simulate the challenge ciphertext as follows:

$$C_{i,1}=T_{\rho^{*}(i)}^{{\hat{r}}_{i}}\cdot (\prod_{j=2}^{n^{*}}{\left(g^{a}\right)}^{M_{i,j}^{*}\cdot \chi_{j}})\cdot {\left(g^{sb_{i}}\right)}^{-{\hat{u}}_{\rho^{*}(i)}}\cdot \prod_{y\in R_{i}}\prod_{j=1}^{n^{*}}{\left(g^{a^{j}\cdot s\cdot \frac{b_{i}}{b_{y}}}\right)}^{M_{y,j}^{*}}$$

and

$$C_{i,2}=g^{-{\hat{r}}_{i}-sb_{i}},\;C_{i,3}=\lambda_{i}^{\prime \prime }$$

B sends the challenge ciphertext $CT_{1}^{*}=\{C,C^{\prime },{\left\{C_{i,1},C_{i,2},C_{i,3}\right\}}_{i\in [1,l^{*}]}\}$ to A.

**Phase2**. A continues to perform a secret key query similar to **Phase1**.

**Guess**. A submits a guess *υ*′ ∈ {0, 1}. If *υ*′ = *υ*, B outputs guess 0 which means $T=e{\left(g,g\right)}^{a^{q+1}\cdot s}$. Otherwise B outputs guess 1 decides that *T* is a random element *R* in $G_{T}$.

Therefore, the advantage of the B in solving the decisional *q*—parallelBDHE problem in a security game is

$$\begin{array}{ll} Adv_{B} & =\frac{1}{2}\left(\text{Pr}[B(\vec{y},T=e{\left(g,g\right)}^{a^{q+1}\cdot s})=0]+\text{Pr}[B(\vec{y},T=R)=0]\right)-\frac{1}{2}\\ & =\frac{1}{2}\left(\frac{1}{2}+\varepsilon +\frac{1}{2}\right)-\frac{1}{2}\\ & =\frac{\varepsilon }{2} \end{array}$$

**Theorem 2**. No polynomial time adversary A can win the trapdoor privacy game with a non-negligible advantage if DBDH assumption holds.

**Proof**. Suppose there is an adversary A which breaks the trapdoor privacy of our scheme with a non-negligible advantage *ε*_*T*_, then we can construct an algorithm B to solve the DBDH problem. Let G be a group of prime order *p* with generator *g* and $e:G\times G\to G_{T}$ be an bilinear map. First, challenger C selects $\beta ,\gamma ,z\in {\mathbb{Z}}_{p}$, *υ* ∈ {0,1} and an element $R\in G_{T}$. We let *Z* = *e*(*g*, *g*)^*βγz*^ if *υ* = 0. Otherwise, *Z* = *R*. Then C gives (*g*, *g*^*β*^, *g*^*γ*^, *g*^*z*^, *Z*) to B. Now let B play the role of challenger in the following security games.

**Setup**. B announces the public key *pk*_*U*_ = *g*^*β*^, *pk*_*O*_ = *g*^*γ*^ with the implicit assumption that *sk*_*U*_ = *β*, *sk*_*O*_ = *γ*.

**Phase1**. A can polynomial query the following oracles:

**Hash Oracle** $O_{H}$: A can ask the random oracle. B maintains a hash list $\langle (pk_{\text{DO}}^{\prime },pk_{\text{DU}}^{\prime },w_{i}),h_{i},e_{i},c_{i}\rangle$ denoted as *L*_*H*_. When

1. If the keyword *w*_*i*_ already appears on the *L*_*H*_ in a tuple $\langle (pk_{O}^{\prime },pk_{U}^{\prime },w_{i}),h_{i},e_{i},c_{i}\rangle$, then B responds with $H(w_{i})=h_{i}\in G$.
2. If the keyword *w*_*i*_ does not exist in the list *L*_*H*_, B throw a random coin *c*_*i*_ ∈ {0,1} so that Pr[*c*_*i*_ = 0] = *σ* and *σ* will be determined later.

If *c*_*i*_ = 0, B computes $h_{i}=g^{z}\cdot g^{e_{i}}\in G$;

If *c*_*i*_ = 1, B computes $h_{i}=g^{e_{i}}\in G$, where $e_{i}\in {\mathbb{Z}}_{p}$ is randomly selected.

Then B adds the tuple $\langle (pk_{O}^{\prime },pk_{U}^{\prime },w_{i}),h_{i},e_{i},c_{i}\rangle$ to the list *L*_*H*_, and returns $H(pk_{O}^{\prime },pk_{U}^{\prime },w_{i})=h_{i}$ to A.

**Trapdoor Querie** $O_{T}$: A gives a tuple $\left(pk_{O}^{\prime },w_{i}\right)$ to ask about the trapdoor. B retrieves $\langle (pk_{\text{DO}}^{\prime },pk_{\text{DU}},w_{i}),h_{i},e_{i},c_{i}\rangle$ from list *L*_*H*_.

If *c*_*i*_ = 0, B claims the failure and output⊥.

If *c*_*i*_ = 1, and hence $h_{i}=g^{e_{i}}\in G$. B computes the trapdoor $T_{w_{i}}$ as

$$\begin{array}{ll} T_{w_{i}} & =e{\left(pk_{O}^{\prime },pk_{U}\right)}^{e_{i}}=e{\left(H(pk_{O}^{\prime },pk_{U},w_{i}),pk_{O}^{\prime }\right)}^{sk_{U}}\\ & =e{\left(g^{\gamma },g^{\beta }\right)}^{e_{i}}=e{\left(H(pk_{O}^{\prime },pk_{U},w_{i}),g^{\gamma }\right)}^{\beta } \end{array}$$

**Index Queries** $O_{I}$: A gives a tuple $\left(pk_{U}^{\prime },w_{i}\right)$ to ask about the index. B randomly chooses $t_{i}\in {\mathbb{Z}}_{p}$ and retrieves $\langle (pk_{\text{DO}},pk_{\text{DU}}^{\prime },w_{i}),h_{i},e_{i},c_{i}\rangle$ from list *L*_*H*_.

If *c*_*i*_ = 0, B claims the failure and output ⊥.

If *c*_*i*_ = 1, let $h_{i}=g^{e_{i}}\in G$. B computes the index *I*_*i*_ as

$$I_{i}=(I_{1,i},I_{2,i})=({\left(pk_{O}\right)}^{e_{i}}\cdot g^{t_{i}},{\left(pk_{U}\right)}^{t_{i}})\;=({\left(g^{\gamma }\right)}^{e_{i}}\cdot g^{t_{i}},{\left(g^{\beta }\right)}^{t_{i}})$$

Finally, B sends trapdoor $T_{w_{i}}$ and index *I*_*i*_ to A.

**Challenge**. A gives two equal length keywords $\left(w_{0}^{*},w_{1}^{*}\right)$, with the restriction that $\left(w_{0}^{*},w_{1}^{*}\right)$ have not been queried to $O_{T}$ nor $O_{I}$. B selects a random bit *υ* ∈ {0,1} and generates the challenge trapdoor $T_{w_{\upsilon }^{*}}$ of keyword $w_{b}^{*}$ and returns it to the A.

If $c_{0}^{*}=1$ and $c_{1}^{*}=1$, B claims the failure and output ⊥.

If $c_{0}^{*}=0$ or $c_{1}^{*}=0$, B selects a random bit *υ* ∈ {0,1} such that $c_{\upsilon }^{*}=0$. B computes the trapdoor $T^{*}=Z\cdot e{\left(g^{\beta },g^{\gamma }\right)}^{e_{i}^{*}}$. If *Z* = *e*(*g*, *g*)^*βγz*^, then $T^{*}=e{\left(g,g\right)}^{\beta \gamma (z+e_{i}^{*})}=e(g^{\beta \gamma },h_{\upsilon^{\prime }})$. Otherwise, T* is a random group element in $G_{T}$.

**Phase2**. Same as **Phase1**, with the restriction $w\neq w_{0}^{*},w_{1}^{*}$.

**Guess**. A will output a guess *υ*′ ∈ {0,1}. If *υ*′ = *υ*, the A wins this game, otherwise A fails.

**Probability Analysis**. Now, we denote by *abort* the event that B aborts during the game. *q*_*T*_ and *q*_*I*_ express the query number of trapdoor oracle $O_{T}$ and index oracle $O_{I}$. There are two cases in which B aborts, as follows.

1. If *c*_*i*_ = 0 when simulating $O_{T}$ and $O_{I}$. Denote it by *abort*_1_. The probability that *abort*_1_ will not occur is $\text{Pr}[\bar{abort_{1}}]={\left(1-\sigma \right)}^{q_{T}+q_{I}}$.
2. If $c_{0}^{*}=1$ and $c_{1}^{*}=1$ in the challenge phase. Denote it by *abort*_2_. The probability that *abort*_2_ will not occur is $\text{Pr}[\bar{abort_{2}}]=1-{\left(1-\sigma \right)}^{2}=2\sigma -\sigma^{2}$. Thence, the probability that B does not terminate the game is

$$\text{Pr}[\bar{abort}]=\text{Pr}[\bar{abort_{1}}]\cdot \text{Pr}[\bar{abort_{2}}]={\left(1-\sigma \right)}^{q_{T}+q_{I}}\cdot (2\sigma -\sigma^{2})$$

When $\sigma =1-\sqrt{q_{T}+q_{I}/q_{T}+q_{I}+2}$, $\text{Pr}[\bar{abort}]$ takes the maximum value

$$\text{Pr}[\bar{abort}]={\left(\frac{q_{T}+q_{I}}{q_{T}+q_{I}+2}\right)}^{q_{T}+q_{I}/2}\cdot \frac{2}{q_{T}+q_{I}+2}$$

which is approximately equal to $\frac{2}{\left(q_{T}+q_{I}\right)}$, and thus non-negligible. Conditioned on that B does not abort, if A succeeds in breaking the trapdoor privacy of our scheme, B also succeeds in telling *Z* = *e*(*g*, *g*)^*βγz*^ or a random element of $R\in G_{T}$. Therefore, the probability that B succeeds in guessing the bit *υ* (and thus solves the DBDH problem) is

$$\begin{array}{ll} \text{Pr}[\upsilon^{\prime }=\upsilon ] & =\text{Pr}[\upsilon^{\prime }=\upsilon |abort]\cdot \text{Pr}[abort]+\;\text{Pr}[\upsilon^{\prime }=\upsilon |\bar{abort}]\cdot \text{Pr}[\bar{abort}]\\ & =\frac{1}{2}(1-\text{Pr}[\bar{abort}])+(\frac{1}{2}+\varepsilon_{T})\cdot \text{Pr}[\bar{abort}]\\ & =\frac{1}{2}+\varepsilon_{T}\cdot \text{Pr}[\bar{abort}] \end{array}$$

If *ε*_*T*_ is non-negligible, so is $\left|\text{Pr}[\upsilon =\upsilon^{\prime }]-\frac{1}{2}\right|$.

## 6 Performance analysis

In this part, we give theoretical and experimental analysis of the proposed scheme.

### 6.1 Theoretical analysis

#### (1) Capability

Here, we give the comparison between our scheme and several related works in terms of features (i.e. Keyword search, Fog computing, Multi-authority, etc.) in Table 1. Observe that, we can see that the schemes [10,22,29] do not have the function of keyword search. Only our scheme and scheme [29] are based on fog computing. With the exception of scheme [10] and our scheme, all users’ attributes in the other schemes are not distributed by multiple authorities. That is to say, multiple authorities design improve the security of the key and reduce the computational pressure of a single authority. Moreover, our scheme and scheme [22] can provide outsourced encryption algorithm. But scheme [22] outsources their computational tasks to the corresponding service providers and does not address the latency response problem. The schemes [16,29] adopts AND-gate access policy and the schemes [18,24] uses a less computationally efficient tree access structure. Our scheme adopts efficient linear secret sharing (LSSS). Fortunately, only our scheme satisfies all properties which makes our scheme more suitable for cloud-fog computing system.

**Table 1:** Features comparison with the main schemes.

Schemes	Keyword search	Fog computing	Multi-authority	Outsourcing encryption	Outsourcing decryption	Access control
[10]	×	×	√	×	×	LSSS
[16]	√	×	×	×	×	AND gate
[18]	√	×	×	×	×	Access tree
[22]	×	×	×	√	√	LSSS
[24]	√	×	×	×	√	Access tree
[29]	×	√	×	×	√	AND gate
Ours	√	√	√	√	√	LSSS

×: This scheme has this function.

√: This scheme does not have this function.

#### (2) Efficiency

In Table 2, we compare the computation cost of our scheme with the schemes [10,16,18,22,24,29] on the key generation, index encryption, trapdoor generation, search over ciphertext, DU-decryption. We mainly consider the time-consuming exponential operation *e* and the bilinear pairing operation *p*. In contrast, the time consumption of the remaining operations is negligible. It can be seen from Table 2 that the literature [10,22,29] does not support keyword search, so there is no computational cost in the index generation, trapdoor generation and search phases. Since our scheme is a multi-authority encryption scheme, the proposed scheme has lower efficiency in the key generation phase than other schemes, but our scheme protects the privacy of the user key and prevents the key from leaking. In the index generation phase, it is obvious that the schemes in [16] is less efficient than our scheme. In addition, the computational complexity of the trapdoor generation and search algorithm are linear with the number of attributes. Our scheme is more efficient than other schemes. It only requires 3 exponential operations and 3 pairs of operations are independent of the number of attributes. In the decryption phase, the efficiency of scheme [10] and our scheme are much higher than that of scheme [18,22,24,29] and the decryption algorithm only needs 1 exponential operation. In general, our proposed scheme has higher search efficiency and lower cost of decryption.

**Table 2:** Comparisons of communication cost.

Schemes	KeyGen	Index	Trapdoor	Search	DU-Decrypt
[10]	(2|*S*| + 5)*e* + *p*	×	×	×	*e*
[16]	(2|*S*| + 3)*e*	(*κ* + 2)*e*	(2|*S*| + 1)*e*	*e* + (|*S*| + 1)*p*	×
[18]	(2|*S*| + 4)*e*	(2*κ* + 1)*e*	(2|*S*| + 4)*e*	|*S*|*e* + (2|*S*| + 1)*p*	*e* + *p*
[22]	(|*S*| + 8)*e* + 5*p*	×	×	×	*p*
[24]	(2|*S*| + 4)*e*	3*e* + (*κ* + 1)*p*	(2|*S*| + 1)*e*	2(|*S*| + 1)*p*	2*p*
[29]	3|*S*|*e* + *p*	×	×	×	*e* + 3*p*
Ours	(4*K* + |*S*_*d*_|)*e*	3*κe*	3*e* + *p*	2*p*	*e*

*p*: the pairing operation.

*e*: the group exponentiation operation in G or $G_{T}$.

*K*: the total number of CAs in the system.

|*S*_*d*_|: the number of attributes managed by the *AA*_*d*_.

*κ*: the number of encrypted keywords in the index.

×: indicates that there is no corresponding algorithm in this scheme.

### 6.2 Experimental analysis

**Fig 4:**
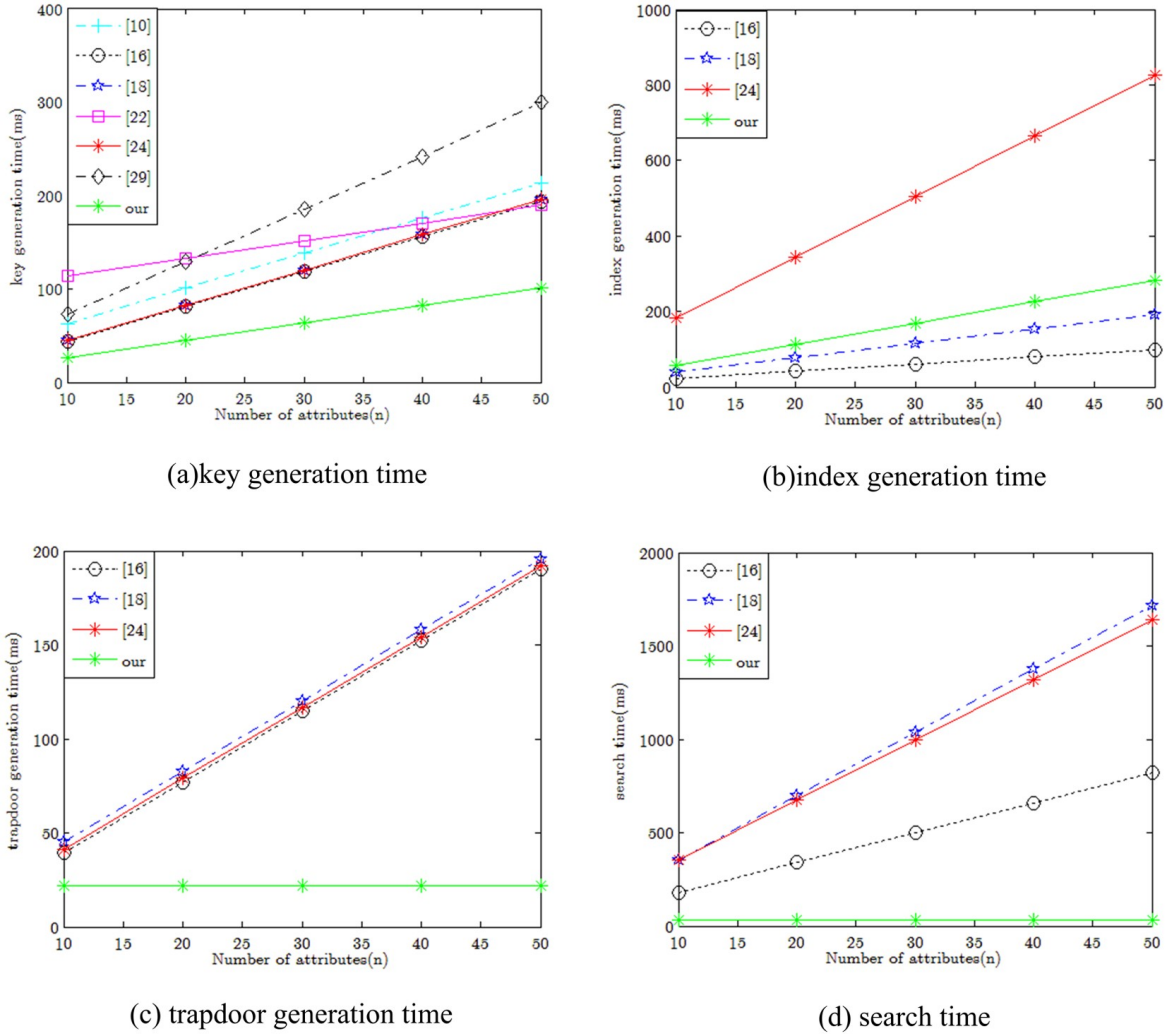
Performance comparison. (A) key generation time (B) index generation time (C) trapdoor generation time (D) search time.

In order to evaluate the practical performance of our scheme, our experiments use the Pairing-Based Cryptography (PBC) library [30]. The environment of the hardware runtime is Intel Core i5-3470 CPU @ 3.20GHz and RAM is 4.00GB. The software runtime environment is JDK 1.7.5, JPBC 2.0.0 and MyEclipse 10. In this section, we describe the efficiency comparison between our scheme and several related literatures [10,16,18,22,24,29]. For the sake of description, we assume the number of user attributes |*S*| ∈ [10, 50] for the keygen, index, trapdoor and search algorithms, which the unified factor is described by the number of attributes. The time is given in milliseconds. From these sub-figures Fig 4(A)~4(D), we show that the number of attributes has an influence on the efficiency of the above four algorithms, respectively.

In Fig 4(A), we show the runtime of the key generation algorithm under different schemes. It can be seen that our solutions are better than all other solutions, and the running time of the schemes [16,18,24] is slightly higher. This is because the theoretical costs of KeyGen algorithm in aforementioned three schemes are (2|*S*| + 3)*e*, (2|*S*| + 4)*e*, (2|*S*| + 4)*e*, respectively. The scheme [29] requires the longest run time and the fastest growth rate. For example, when setting |*S*| = *n* = 30, the time required for these seven programs is 138.934ms, 118.566ms, 120.448ms, 151.836, 120.448ms, 185.444ms and 63.988ms, respectively. The growth of scheme [22] is relatively flat, and at |*S*| = 10, the time of key generation is the longest in all relevant literature.

Schemes in [10,22,29] do not have the function of keyword search, there are no index generation, trapdoor generation and ciphertext retrieval curve for the scheme of [10,22,29] in Fig 4(B)~4(D). In Fig 4(B) we show the time of index generation in the encryption algorithm. By changing the value of *n* from 10 to 50, we notice that the computational burden of our scheme is slightly higher than that of schemes [16] and [18]. However, since the encryption algorithm is a one-time cost, it does not affect the user’s search experience. Therefore, its communication cost is acceptable in practical applications.

In Fig 4(C), we present the time cost of the trapdoor generation algorithm in all schemes. Schemes [10], [18] and [24] have only subtle differences in the trapdoor generation phase and the time spent is linearly increasing with the number of attributes. However, our scheme is a tiny constant that only needs 3*e* + *p* operations, regardless of the number of attributes.

Focusing on the search algorithm, we also tested the time spent in the ciphertext retrieval phase. In this experiment, the calculation cost of the scheme [18] and [24] were the highest, and the scheme [18] (or the scheme [24]) needs |*S*|*e* + (2|*S*| + 1)*p* (or (2|*S*| + 1)*p*) operations. The computational cost of these three schemes [16,18,24] grows linearly with the number of attributes. While our scheme is optimal in all schemes. This is because the time of ciphertext search is independent of variable *n*, our scheme just needs 2*p* operations.

From Fig 4 we can see that the actual experimental simulation is completely consistent with the theoretical analysis. Therefore, our scheme is feasible and efficient in practical environment.

## 7 Conclusions

In this paper, we have presented a searchable encryption scheme based on cloud-fog computing, in which end users could greatly reduce the computational and storage burden by outsourcing part of the operation to the fog node. Specially, with the application scenarios named PHRs, the scheme enables patients to safely store PHRs shared with their doctor or family on a cloud server, while the patient’s personal information remains confidential. Furthermore, our solution supports keyword search and fine-grained access control to further narrow down the search scope and avoid unauthorized user’s access. Finally, our scheme is proven IND-sCP-CCA secure and trapdoor privacy secure. As part of our future work, we will continue to explore expressive search, fuzzy keyword search, multi-dimensional scope query or no central authority in the system, and so on. Meanwhile, we also need to further improve the efficiency of our system so that it can be applied to various programs.

## Supporting information

S1 File The runtime of cryptographic operations. (DOC)

S2 File The summary of the new notations. (DOC)
