# Supplementary material for: A searchable personal health records framework with fine-grained access control in cloud-fog computing
Source: PLoS One. 2018 Nov 29;13(11):e0207543. doi: 10.1371/journal.pone.0207543 (PMC6264141; doi:10.1371/journal.pone.0207543)
Supplement: S1 File — (DOC) [file pone.0207543.s001.doc]

**S1 Appendix**

The experimental part of this paper refers to the results in the Pairing Based Cryptography (PBC) library.

**The runtime of cryptographic operations**

| Operating | Ad | Ne | Mu | In | Ex | Add | Neg | PM | Mul | Inv | Exp | P |
| --- | --- | --- | --- | --- | --- | --- | --- | --- | --- | --- | --- | --- |
| Times/ms | 0.001 | 0.000 | 0.001 | 0.004 | 0.067 | 0.038 | 0.001 | 8.006 | 0.013 | 0.041 | 1.882 | 16.064 |

^1^Ad: an addition operation in;

^2^Ne: the inverse in addition operation in;

^3^Mu: a multiplication operation in;

^4^In: the inverse in multiplication operation in;

^5^Ex: an exponentiation operation in;

^6^Add: an addition operation in;

^7^Neg: the inverse in addition operation in;

^8^PM: a point multiplication operation in;

^9^Mul: a multiplication operation in.

^10^Inv: the inverse in multiplication operation in .

^11^Exp: an exponentiation operation in.

^12^P: an bilinear pairings operation in .
