# Supplementary material for: A searchable personal health records framework with fine-grained access control in cloud-fog computing
Source: PLoS One. 2018 Nov 29;13(11):e0207543. doi: 10.1371/journal.pone.0207543 (PMC6264141; doi:10.1371/journal.pone.0207543)
Supplement: S2 File — (DOC) [file pone.0207543.s002.doc]

**Table. The summary of the new notations.**

| **Symbols** | **Descriptions** |
| --- | --- |
| $S_{d}$ | attribute set managed by attribute authority$AA_{d}$ |
| $k$ | index of the CA |
| $K$ | the total number of CAs in the system |
|  | set of different CAs |
| $d$ | index of the AA |
| $D$ | the total number of AAs in the system |
|  | set of different AAs |
|  | attribute universe |
